# Normally-off β-Ga_2_O_3_ MOSFET with an Epitaxial Drift Layer

**DOI:** 10.3390/mi13081185

**Published:** 2022-07-27

**Authors:** Chan-Hee Jang, Gökhan Atmaca, Ho-Young Cha

**Affiliations:** School of Electrical and Electronic Engineering, Hongik University, Mapo-gu, Seoul 04066, Korea; booj0429@nate.com (C.-H.J.); gokhanatmaca@kuark.org (G.A.)

**Keywords:** accumulation channel, β-Ga_2_O_3_, epitaxial drift layer, metal-oxide-semiconductor field-effect transistor, normally-off

## Abstract

A normally-off β-Ga_2_O_3_ metal-oxide-semiconductor field-effect transistor (MOSFET) is proposed using a technology computer-aided design (TCAD) device simulation, which employs an epitaxial drift layer grown on an *n*-type low-doped body layer. The low-doped body layer under the MOS gate enabled normally-off operation, whereas the epitaxial drift layer determined the on-resistance and breakdown characteristics. The effects of the doping concentration of each layer and thickness of the drift channel layer on the device characteristics were investigated to design a device with a breakdown voltage of 1 kV. A threshold voltage of 1.5 V and a breakdown voltage of 1 kV were achieved by an *n*-type body layer with a doping concentration of 1 × 10^15^ cm^−3^ and an *n*-type drift layer with a doping concentration of 3 × 10^17^ cm^−3^, a thickness of 150 nm, and a gate-to-drain distance of 9.5 μm; resulting in an on-resistance of 25 mΩ·cm^2^.

## 1. Introduction

Wide bandgap (WBG) semiconductors, such as GaN, SiC, and Ga_2_O_3_, have been intensively studied to overcome the limitations of Si technology [1,2,3] for the development of next-generation power switching devices. Ga_2_O_3_ has a wider energy bandgap (4.5–4.9 eV) than GaN and SiC, with a significantly higher critical electric field of approximately 6–8 MV/cm [2,3,4,5]. Among the various polymorphs, monoclinic β-Ga_2_O_3_ is the most stable polymorph [6,7,8,9,10]. Furthermore, Baliga’s figure of merit (BFOM) of β-Ga_2_O_3_ is significantly higher than those of GaN and SiC, making it a promising material for high-power switching applications [10]. However, the absence of *p*-type doping technology for β-Ga_2_O_3_ is disadvantageous for the implementation of power switching devices [11,12,13,14,15,16,17]. Although several studies have reported the normally-off operation of β-Ga_2_O_3_ field-effect transistors (FET), the experimental results are still far off from the theoretical limits of the material [15,16,17,18].

Chabak et al., demonstrated enhancement-mode FETs using a wrap-gate fin structure in 2016 [15] and a gate recess process in 2018 [16]. In 2017, Wong et al., reported that the utilization of an unintentionally doped β-Ga_2_O_3_ channel in MOSFET was able to completely deplete the channel electrons at a gate voltage (V_GS_) of 0 V, resulting in a positive threshold voltage [19]. In 2019, Singh et al., proposed a T-shaped recessed gate β-Ga_2_O_3_ MOSFET to achieve a normally-off operation [16]. The T-shaped recessed gate depleted the channel at a gate bias of 0 V, where the gate oxide (Al_2_O_3_) thickness was 20 nm, gate recess depth was 250 nm, and thickness of the active channel under the recess region was 30 nm [17]. The maximum drain current was 40 mA/mm at V_GS_ = +8 V due to the limited channel thickness required to achieve a positive threshold voltage [17].

In this study, we propose a recessed β-Ga_2_O_3_ MOSFET with an epitaxial drift layer on top of a low-doped body layer to overcome the trade-off relationship between the threshold voltage and on-current density. The proposed structure does not require precise control of recess depth. Moreover, the threshold voltage could be independently controlled by the drift layer. The output and transfer characteristics of the proposed device were validated using Silvaco ATLAS technology computer-aided design (TCAD) simulation. After investigating the effects of doping concentration on the body layer and additional design parameters of the drift layer, a normally-off MOSFET structure was designed to achieve a breakdown voltage of 1 kV.

## 2. Simulation and Device Structure

Two-dimensional (2D) device simulations were performed in a Silvaco ATLAS TCAD environment using several physical models, including a drift-diffusion transport model, Fermi–Dirac statistics, concentration and temperature-dependent analytical mobility model, Shockley–Read–Hall recombination model, and an impact ionization model [20,21,22,23,24,25,26]. The material and physical model parameters used in the TCAD simulations are presented in Table 1. Although the simulation process could have been further optimized by employing more comprehensive models [27], the classical models provided by TCAD are sufficient to validate the proposed concept.

### 2.1. Mobility Model

The mobility model used in the simulation included concentration and temperature-dependent relationships based on an analytical function of Caughey–Thomas’ work [25], which is given by:

(1) $$\mu_{n0}=\mu_{min}{\left(\frac{T_{L}}{300}\right)}^{\alpha }+\frac{\mu_{max}{\left(\frac{T_{L}}{300}\right)}^{\beta }-\mu_{min}{\left(\frac{T_{L}}{300}\right)}^{\beta }}{1+{\left(\frac{T_{L}}{300}\right)}^{\gamma }{\left(\frac{N_{D}}{N_{ref}}\right)}^{\delta }}\;$$

where *α*, *β*, γ, and *δ* are material-dependent coefficients [20], *N_D_* is the impurity concentration, and *T_L_* is the temperature in Kelvins. Using the experimental data [28,29], these parameters were determined to be *α* = 0, *β* = 0, *γ* = 0, *δ* = 0.8, *N_ref_* = 1.0 × 10^18^ cm^−3^, and *T_L_* = 300 K.

### 2.2. Impact Ionization Model

Selberherr’s model, which is a modification of Chynoweth’s law, has been widely used to predict the breakdown characteristics of wide-bandgap semiconductors [20,21]. The impact ionization coefficient (α_n_) is given by

(2) $$\alpha_{n}=A_{N}\exp\left[-\frac{B_{N}}{E}\right]$$

where *A_N_* and *B_N_* are the material coefficients and E is the electric field. In this study, *A_N_* = 2.16 × 10^6^ cm^−1^ and *B_N_* = 1.77 × 10^7^ V/cm were used while considering the crystal direction of β-Ga_2_O_3_ in the [010] direction and a critical electric field of approximately 5 MV/cm [11,20,21].

### 2.3. Shockley–Read–Hall Recombination

In our simulations, the recombination rate was obtained using the Shockley–Read–Hall recombination model [26]:

(3) $$R_{SRH}=\frac{pn-n_{ie}^{2}}{\begin{array}{c} \tau_{p0}\left[n+n_{ie}e^{\left(\frac{E_{trap}}{kT_{L}}\right)}\right]+\tau_{n0}\left[p+n_{ie}e^{\left(\frac{-E_{trap}}{kT_{L}}\right)}\right] \end{array}}\;$$

where *n*, *p*, and *n_ie_* are the electron, hole, and intrinsic carrier concentrations, respectively, and *k* and *T_L_* are the Boltzmann constant and lattice temperature, respectively. *E_trap_* is the difference between the trap energy level and the intrinsic Fermi level, and *τ_n_*_0_ and *τ_p_*_0_ are the electron and hole lifetimes, respectively, which are used as 1.2 × 10^−8^ s.

### 2.4. Device Structure

**Figure 1:**
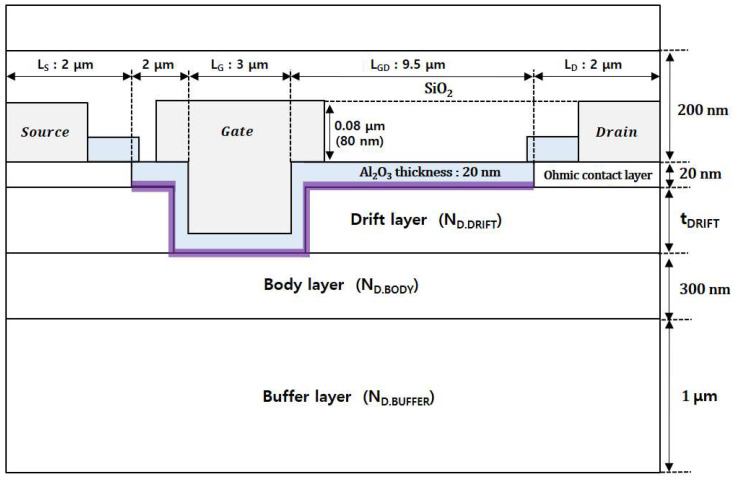
Cross-sectional schematic of recessed β-Ga_2_O_3_ MOSFET with a dual epitaxial structure.

Figure 1 shows a cross-sectional schematic of the β-Ga_2_O_3_ MOSFET proposed in this study. The epitaxial structure consisted of a 20 nm thick ohmic contact layer with an *n*-type doping concentration of 1 × 10^20^ cm^−3^, an *n*-type drift layer, a 300 nm thick low-doped *n*-type body layer, and a 1 μm thick buffer layer with an *n*-type doping concentration of 1 × 10^12^ cm^−3^. In this study, a highly doped ohmic contact layer was employed instead of an ion-implantation process. The structural variables investigated in this study were the thickness (t_DRIFT_) and doping concentration (N_D.DRIFT_) of the drift layer and the doping concentration (N_D.BODY_) of the body layer. A highly doped ohmic contact layer is etched between the source and drain contacts. The gate region was etched down to the body layer to achieve normally-off characteristics. A 20 nm-thick gate oxide (Al_2_O_3_) layer was used, and its interface charges were considered during the simulation.

**Figure 2:**
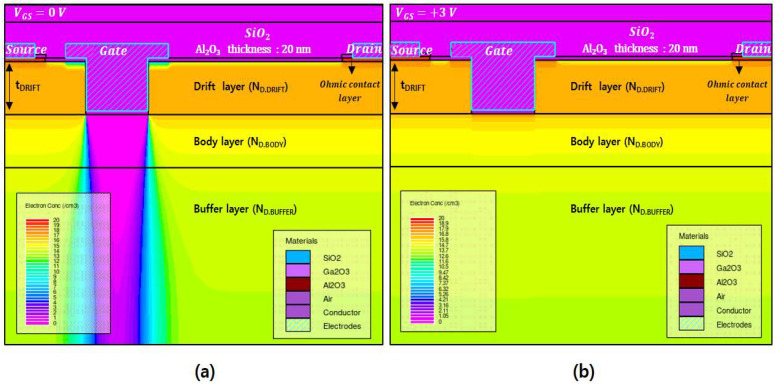
Electron density distributions simulated with a gate voltage of (**a**) V_GS_ = 0 V and (**b**) V_GS_ = +3 V.

Figure 2a,b show the electron density distributions at V_GS_ = 0 V and +3 V, respectively, which were simulated using the variables N_D.DRIFT_ = 3 × 10^17^ cm^−3^, t_DRIFT_ = 300 nm, and N_D.BODY_ = 1 × 10^15^ cm^−3^. For V_GS_ = 0 V, the electrons in the region under the gate were completely depleted, which blocked the flow of current, confirming the normally-off characteristics. For V_GS_ = +3 V, the depletion region under the gate disappeared, creating an electron accumulation channel layer and allowing for current flow.

## 3. Results and Discussions

### 3.1. Effects of Al_2_O_3_/β-Ga_2_O_3_ Interface Charge

**Figure 3:**
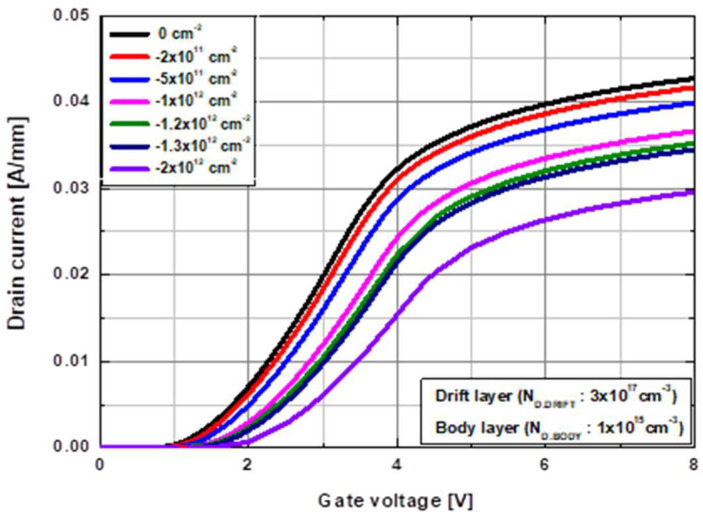
Shift in threshold voltage with different interface charge densities at the Al_2_O_3_/β-Ga_2_O_3_ interface.

Previous studies have reported the presence of negative interface charges at the Al_2_O_3_/β-Ga_2_O_3_ interface with a density in the range of 1 × 10^12^ to 4 × 10^12^ cm^−2^ [18,21,23,30,31]. In this section, the effects of charge density at the Al_2_O_3_/β-Ga_2_O_3_ interface are investigated, where the negative interface charge density varied from 0 to 2 × 10^12^ cm^−2^. The transfer characteristics simulated at a drain voltage (V_DS_) of 5 V as a function of the interface charge density are shown in Figure 3. A positive shift in the threshold voltage was observed with a reduction in drain current density as the negative interface charge density increased. Therefore, based on these prior experimental reports [18,20], a negative interface charge density of 1 × 10^12^ cm^−2^ was selected for the simulations.

### 3.2. Effects of Doping Concentrations in Body and Drift Layer

**Figure 4:**
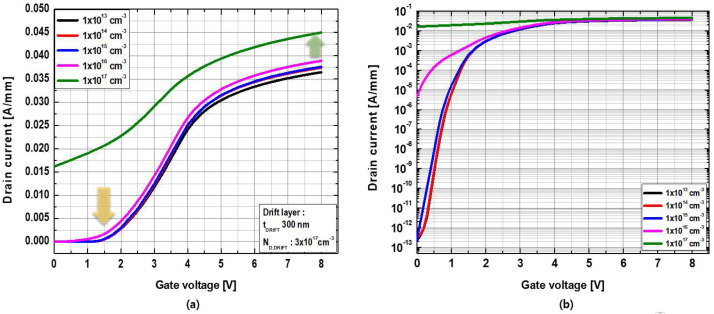
(**a**) Linear and (**b**) logarithmic transfer characteristics at V_DS_ = +5 V as a function of the doping concentration of the body layer.

Initially, the effects of the doping concentration of the body layer (N_D.BODY_) on the threshold voltage were investigated. N_D.BODY_ varied from 1 × 10^13^ cm^−3^ to 1 × 10^17^ cm^−3^, while the drift layer had a thickness of t_DRIFT_ = 300 nm and a doping concentration of N_D.DRIFT_ = 3 × 10^17^ cm^−3^. Figure 4a,b show the linear and logarithmic transfer characteristics at V_DS_ = 5 V as a function of N_D.BODY_, respectively. A significant negative shift in the threshold voltage was observed when N_D.BODY_ was equal to or greater than 1 × 10^16^ cm^−3^, resulting in normally-on characteristics, whereas only a negligible difference was observed when N_D.BODY_ was equal to or less than 1 × 10^15^ cm^−3^. Therefore, to design a normally-off device, N_D.BODY_ = 1 × 10^15^ cm^−3^ was selected for the simulations.

**Figure 5:**
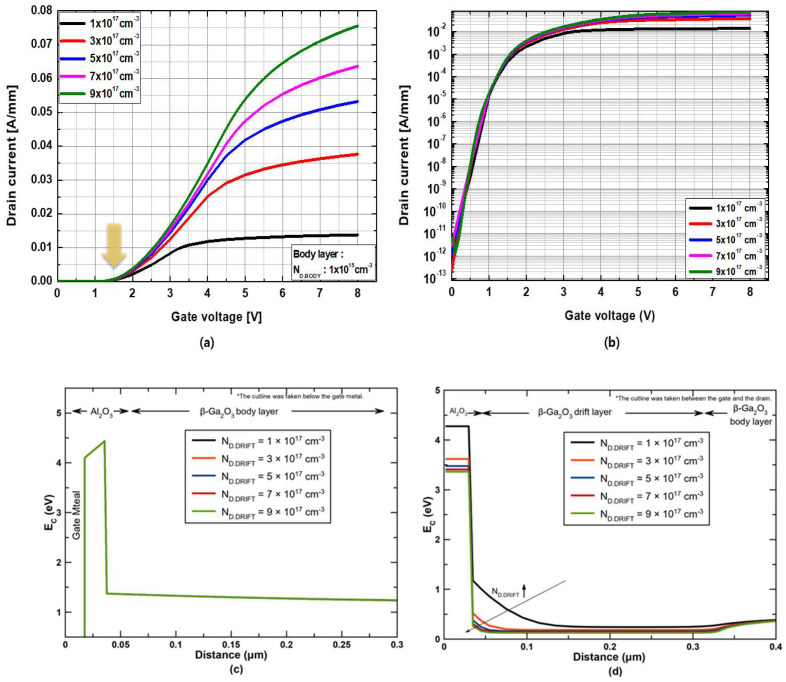
(**a**) Linear and (**b**) logarithmic transfer characteristics at V_DS_ = +5 V as a function of the doping concentration of the drift channel layer. Conduction band energy diagrams as a function of N_D.DRIFT_ along the vertical direction (**c**) under the gate metal and (**d**) in the region between the gate and drain at zero gate bias condition.

Additionally, the effects of the doping concentration of the drift layer (N_D.DRIFT_) on the drain current density were investigated, where N_D.DRIFT_ varied from 1 × 10^17^ cm^−3^ to 9 × 10^17^ cm^−3^ with a fixed body doping concentration of N_D.BODY_ = 1 × 10^15^ cm^−3^. The drift layer thickness was t_DRIFT_ 300 nm. The transfer characteristics as a function of N_D.DRIFT_ are shown in Figure 5a,b. It is evident that the drain current density increases with an increase in N_D.DRIFT_, whereas the threshold voltage remains the same because it is determined by the recessed MOS region on the body layer. The normally-off characteristics were maintained even at N_D.DRIFT_ = 9 × 10^17^ cm^−3^. The threshold voltage was 0.8 V at 1 μA/mm and 1.5 V at 1 mA/mm. Figure 5c shows the conduction band energy diagrams as a function of N_D.DRIFT_ along the vertical direction below the gate metal, and it is obvious that increasing N_D.DRIFT_ does not change the conduction band energy such that the threshold voltage remains the same regardless of N_D.DRIFT_. On the other hand, Figure 5d shows the conduction band energy diagrams as a function of N_D.DRIFT_ along the vertical direction in the region between the gate and drain. It can be seen that the depletion width in the β-Ga_2_O_3_ drift layer is reduced when increasing the N_D.DRIFT_, leading to a higher drain current.

### 3.3. Effects of Drift Layer Thickness

#### 3.3.1. Transfer and Output Characteristics

**Figure 6:**
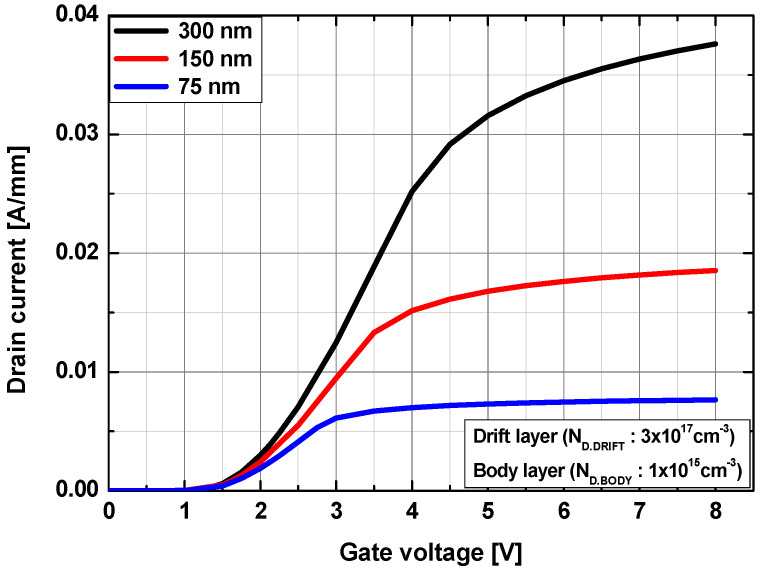
Transfer characteristics at V_DS_ = +5 V as a function of the thickness of the drift channel layer. The drift channel layer has a doping concentration of 3 × 10^17^ cm^−3^.

**Figure 7:**
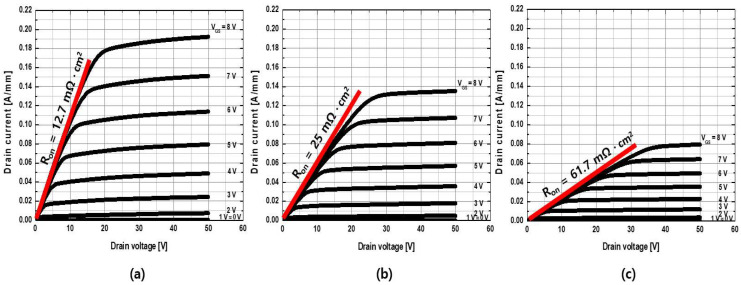
Output current–voltage characteristics for a drift channel layer thickness of (**a**) 300 nm, (**b**) 150 nm, and (**c**) 75 nm. The drift channel layer has a doping concentration of 3 × 10^17^ cm^−3^.

To investigate the effects of the thickness of the drift layer (t_DRIFT_), the doping concentrations of the body and drift channel layers were fixed as N_D.BODY_ = 1 × 10^15^ cm^−3^ and N_D.DRIFT_ = 3 × 10^17^ cm^−3^, respectively. The t_DRIFT_ was varied to 75, 150, and 300 nm. As shown in Figure 6, the drain on-current density decreased with a decrease in t_DRIFT_, while the threshold voltage remained constant as the series resistance of the drift layer increased with a decrease in the thickness. The output current–voltage characteristics are compared in Figure 7. The maximum drain current density (I_D.MAX_) and on-resistance (R_on_) for the thicknesses of t_DRIFT_ = 300, 150, and 75 nm were I_D.MAX_ = 190, 136, and 80 mA/mm, respectively, and R_on_ = 12.7, 25, and 61.7 mΩ cm^2^, respectively.

#### 3.3.2. Breakdown Characteristics

**Figure 8:**
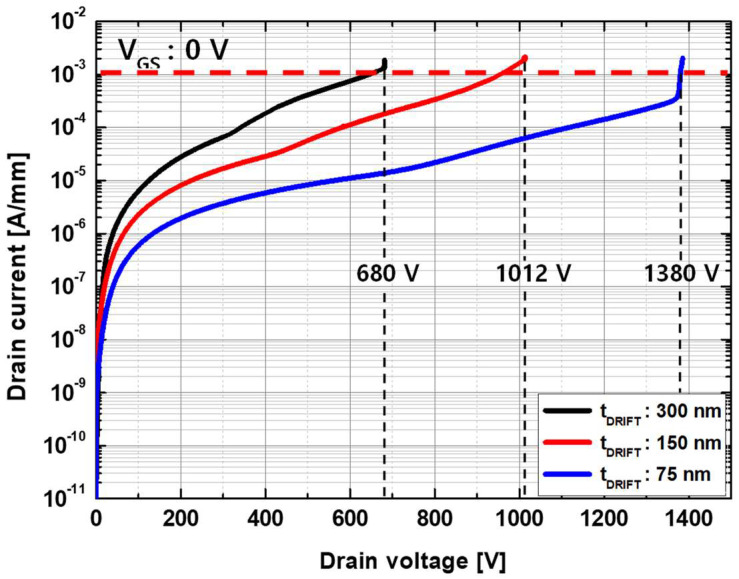
Breakdown voltage characteristics simulated at V_GS_ = 0 V as a function of the drift channel layer thickness. The drift channel layer has a doping concentration of 3 × 10^17^ cm^−3^.

**Figure 9:**
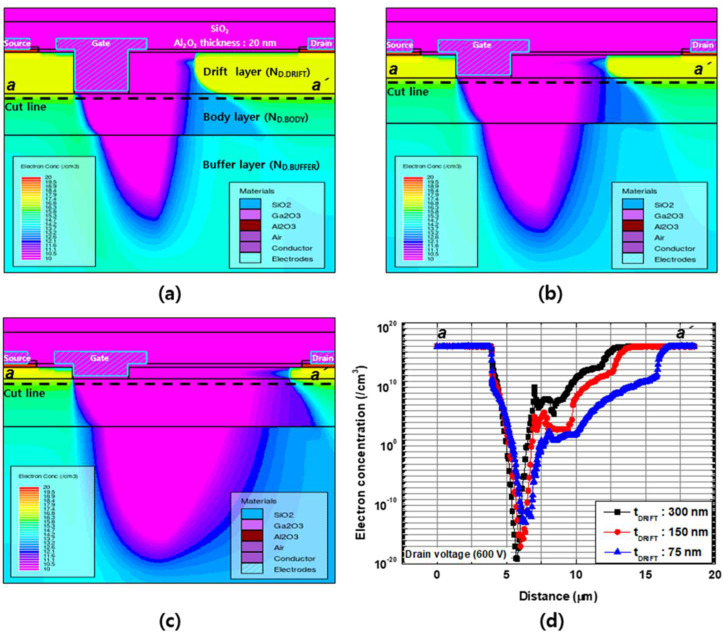
Electron density distributions simulated with V_GS_ = 0 V and V_DS_ = + 600 V for a drift channel layer thicknesses of (**a**) 300 nm, (**b**) 150 nm, and (**c**) 75 nm. (**d**) Electron density distribution along the cutline between a and a’.

**Figure 10:**
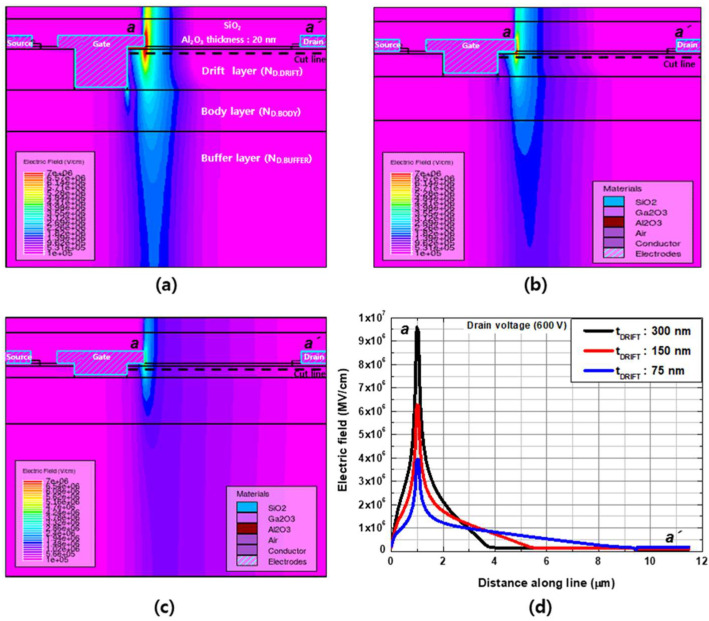
Electric field distributions simulated with V_GS_ = 0 V and V_DS_ = +600 V for a drift channel layer thicknesses of (**a**) 300 nm, (**b**) 150 nm, and (**c**) 75 nm. (**d**) Electric field distribution along the cutline between a and a’.

**Figure 11:**
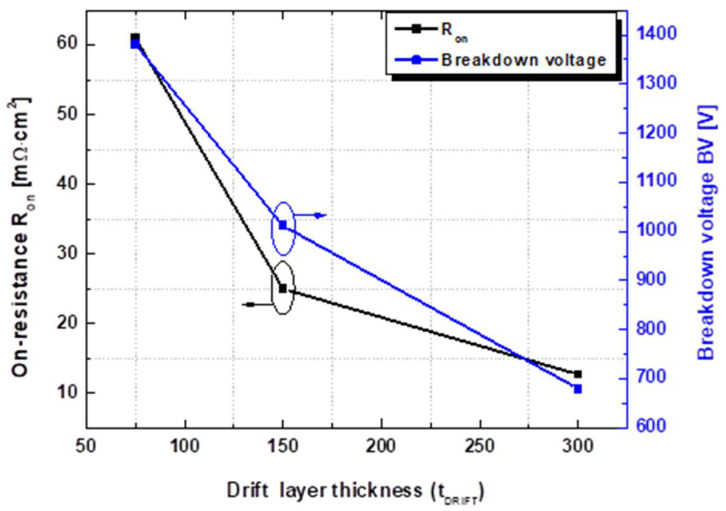
On-resistance and breakdown voltage characteristics as a function of drift layer thickness. The drift layer has a doping concentration of 3 × 10^17^ cm^−3^.

Breakdown characteristics with different drift layer thicknesses were simulated at V_GS_ = 0 V, and the results are compared in Figure 8. The catastrophic breakdown voltages were 680, 1012, and 1380 V for the thickness values of t_DRIFT_ = 300, 150, and 75 nm, respectively. With the same doping concentration of the drift layer, the breakdown voltage exhibited a significant dependence on t_DRIFT_. The electron density and electric field distributions for different t_DRIFT_ values were examined to investigate the reasons for this. Figure 9 and Figure 10 show the electron density and electric field distributions simulated at V_DS_ = 600 V for different t_DRIFT_ values, and the electron concentration and electric field distributions along the cutline from a to a’ are plotted in Figure 9d and Figure 10d, respectively. As shown in Figure 9d and Figure 10d, the depletion region extended towards the drain side with decreasing thickness, resulting in a lower peak electric field near the gate. This is because the total number of electrons depleted by a given gate voltage is the same for all cases. Therefore, the thinner drift layer had a longer depletion edge. Consequently, a higher breakdown voltage can be achieved with a thinner drift layer. The tradeoff relationship between R_on_ and the breakdown voltage as a function of the drift layer thickness is shown in Figure 11.

In summary, using a body layer with a doping concentration of 1 × 10^15^ cm^−3^ and a drift layer with a doping concentration of 3 × 10^17^ cm^−3^, a thickness of 150 nm, and a gate-to-drain distance of 9.5 μm resulted in a threshold voltage of 0.8 V at 1 μA/mm, a breakdown voltage of ~1 kV, and an on-resistance of 25 mΩ·cm^2^.

## 4. Conclusions

A normally-off β-Ga_2_O_3_ MOSFET structure was proposed, which employed an epitaxial drift layer in conjunction with a recessed MOS gate. A positive threshold voltage was achieved by employing a low-doped *n*-type body layer, which led to the formation of an electron-accumulation channel layer. An additional drift layer grown on top of the body layer is crucial for determining the on-resistance and breakdown voltage characteristics. The proposed dual epitaxial structure enables normally-off operation without employing an ion implantation process. Considering the difficulty of *p*-type ion implantation or epitaxial growth with β-Ga_2_O_3_, the proposed structure is a promising candidate for the implementation of a normally-off β-Ga_2_O_3_ FET.

## Figures and Tables

**Table 1:** Material and physical model parameters used for TCAD simulations [20,21,22,23,24,25,26,28,29].

Material Parameters	
Affinitivity	4.0 eV
Band gap (300 K)	4.8 eV
Permittivity	10.2
Mobility Model	
*μ_min_*	20 cm^2^/Vs
*μ_max_*	155 cm^2^/Vs
*α*	0
*β*	0
*γ*	0
*δ*	0.8
*N_ref_*	1.0 × 10^18^ cm^−3^
*T_L_*	300 K
Impact Ionization Model	
*A_N_*	2.16 × 10^6^ cm^−1^
*B_N_*	1.77 × 10^7^ V/cm
Shockley–Read–Hall Recombination Model	
*τ_n_*_0_	1.2 × 10^−8^ s
*τ_p_*_0_	1.2 × 10^−8^ s
